# Association between health behaviors and mood disorders among the elderly: a community-based cohort study

**DOI:** 10.1186/s12877-019-1079-1

**Published:** 2019-02-28

**Authors:** Tzu-Jung Tseng, Yi-Syuan Wu, Jia-Hong Tang, Yen-Hui Chiu, Yu-Ting Lee, I-Chun Fan, Ta-Chien Chan

**Affiliations:** 1 Research Center for Humanities and Social Sciences, Academia Sinica, Taipei, Taiwan; 2 Graduate Institute of Life Sciences, National Defense Medical Center, Taipei, Taiwan; 3 Institute of Statistical Science, Academia Sinica, Taipei, Taiwan; 4 Department of Education and Research, Taipei City Hospital, Taipei, Taiwan; 5 Institute of History and Philology, Academia Sinica, Taipei, Taiwan; 6 Institute of Public Health, School of Medicine, National Yang-Ming University, Taipei, Taiwan

**Keywords:** Elderly health examination database, Socio-economic status, Health behaviors, Mental health, Mood disorders

## Abstract

**Background:**

According to a WHO report, nearly 15% of adults aged 60 and over suffer from a mental disorder, constituting 6.6% of the total disability for this age group. Taipei City faces rapid transformation towards an aging society, with the proportion of elderly in the total population rising from 12% in 2008 to 16% in 2016. The aim of this study is to identify the prevalence of mental disorders among the elderly in Taipei City and to elucidate risk factors contributing to mental disorders.

**Methods:**

The elderly health examination database was obtained from the Department of Health, Taipei City government, from 2005 to 2012. A total of 86,061 people underwent publicly funded health examinations, with 348,067 visits. Each year, there are around 43,000 elderly persons in Taipei City using this service. We used a mental health questionnaire including five questions to estimated relative risks among potential risk factors with the generalized estimating equations (GEE) model to measure the mental health status of the elderly. Mood disorders were measured with the Brief Symptom Rating Scale (BSRS-5) questionnaire. Age, education level, gender, marital status, living alone, drinking milk, eating vegetables and fruits, long-term medication, smoking status, frequency of alcohol consumption, frequency of physical activity, BMI, and number of chronic diseases were included as covariates.

**Results:**

The results show that being male (odds ratio (OR) 0.57; 95% CI = 0.56, 0.59), higher education (OR 0.88; 95% CI = 0.82, 0.95), no long-term medication (OR 0.57; 95% CI = 0.56, 0.58), and exercising three or more times per week (OR 0.94; 95% CI = 0.91, 0.98) were all positively correlated with better emotional status. However, being divorced (OR = 1.22, 95% CI = 1.09, 1.36), not drinking milk (OR = 1.12, 95% CI = 1.09, 1.14), not eating enough vegetables and fruits every day (OR = 1.78, 95% CI = 1.73, 1.83), daily smoking (OR = 1.15, 95% CI = 1.01, 1.32), and having more chronic diseases (OR = 1.02, 95% CI = 1.01, 1.03) were all correlated with poor mental status among the elderly.

**Conclusions:**

The findings of this research can both estimate the prevalence of mood disorders at the community level, and identify risk factors of mood disorders at the personal level.

## Background

The ageing population is growing rapidly. The World Health Organization (WHO) estimated that the proportion of the elderly population in the world will jump from 12 to 22% between 2015 and 2050. Over 20% of elderly persons have suffered from mental or neurological disorders [1]. In addition, several community-based cohort studies have found that mood disorders are an important risk factor for suicide in the elderly, and significantly reduce the quality of life and increase disease burden [2]. According to a National Health Service (NHS) report in the UK, half of adults (7.7million) aged 55 and over have experienced common mental health problems [3]. Therefore, the UK government established a plan to help the elderly through general practitioners (GP) and a complete community care system to detect the signs of mood disorders early [4, 5]. In the United States, 18.29% of adults (nearly 43.7 million Americans) struggle with mental health problems annually [6]. Additionally, the proportion of older adults who experience mood disorders is 6.8% [7]. A study with a sample of 3142 older men and women (aged 65–84 years) indicated that one in two individuals had experienced mental disorders in their lifetime, one in three within the past year, and nearly one in four currently had a mental disorder [8]. In Asia, including the elderly, about 8.8% of people aged above 20 suffer mood disorders in Japan [9]. With a growing proportion of older adults in Japan, the number of older adult patients (≥65 years old) with mood or anxiety disorders has also been increasing, reaching 340,000 in 2014 [10]. In the context of this rapid population aging, there is a growing interest in promoting healthy aging, including the mental health of the elderly.

There are multiple risk factors for mental health problems in the elderly, such as demographic characteristics, e.g., being female, unmarried, living alone, or children not being around [11]. Socio-economic indicators such as low household income [11], low education level [12], and other individual characteristics like chronic diseases, lack of participation in community sports or physical activities [13], physical illness, and poor health status are all associated with higher rates of mood disorders [14]. In addition, one study found that regular physical activity in midlife is significantly associated with reduced depressive symptoms 25 years later [15]. Previous research also indicated that physical activity is a protective factor for mental health in older adults [16]. However, light physical activity, independent of moderate to vigorous physical activity, is associated with fewer depressive symptoms [17]. In summary, the past studies pointed out that being female [11], divorced [11], living alone [11], low education level [12], not doing physical activity [13, 15], having chronic diseases, and bad health status are correlated with mood disorder in the elderly [14]. However, the correlation between smoking behavior and mood disorder in the elderly has diverse conclusions in the literature [18, 19]. Some health behaviors considered in this study such as drinking alcohol, eating vegetables and fruits, and drinking milk are not mentioned in past studies. In addition, this study collected a wide spectrum of the elderly population, including healthy and sick elderly in the communities, and provided the opportunity to identify the changeable behaviors in daily life.

Preventive intervention can reduce the disease burden of mood disorders. Previous studies have indicated that prevention and intervention in the early stages are very effective to reduce severity and prevent secondary disorders, such as degradation of cognitive functions [20]. Sarris and colleagues [21] provide a nexus between public health promotion and clinical treatments, involving the application of environmental and behavioral interventions to enhance physical and mental well-being, calling it “Life Medicine.” However, these studies recruited a wide age range of subjects, or focused on the disease of depression itself. As far as we know, there is little information on how to use community health examinations to identify mood disorders or emotional problems in the elderly [18]. In addition, few studies have large samples and discuss health in the elderly in a community with a longitudinal cohort.

In this study, we applied the Taipei City Elderly Health Examination Database for a total of eight years of follow-up data to identify possible risk factors early. The database included not only the basic physical characteristics of the individual, but also variables of healthy behavior, for example, the frequency of smoking, drinking alcohol, frequency of physical activity, and the number of underlying diseases and drug history. The primary objective of this study was to elucidate the potential risk factors associated with mood disorders through using BSRS-5 (5-item Brief Symptom Rating Scale) scores [22]. The second aim of this study was to find risk and protective factors with mood disorders, so as to be able to provide health policy makers suggestions for health education intervention to reduce the risk of mood disorders in the elderly.

## Methods

### Ethics

This study was designed as a retrospective longitudinal cohort study. Every enrolled participant needed to fill out an informed consent form to authorize the Taipei City Government to process health examination data for the research purpose [23]. Data are stored centrally in the Taipei Geriatric Health Examination Database and are de-identified before release. This study was approved by the Institutional Review Board (IRB) on Biomedical Science Research, Academia Sinica (AS-IRB02–104182).

There are two phases for the elderly to join this publicly funded health examination. The essential two criteria are age > =65 years and having one’s registered residence in Taipei City. Phase one is for aborigines, those living alone, low-income households and disabled people. Registration is by phone and the internet during the last week of February each year. Phase two is open to all elderly persons who are eligible for the health examination that register by telephone, internet and on-site registration. All participants can receive the health examination once annually.

### Data source

**Fig. 1:**
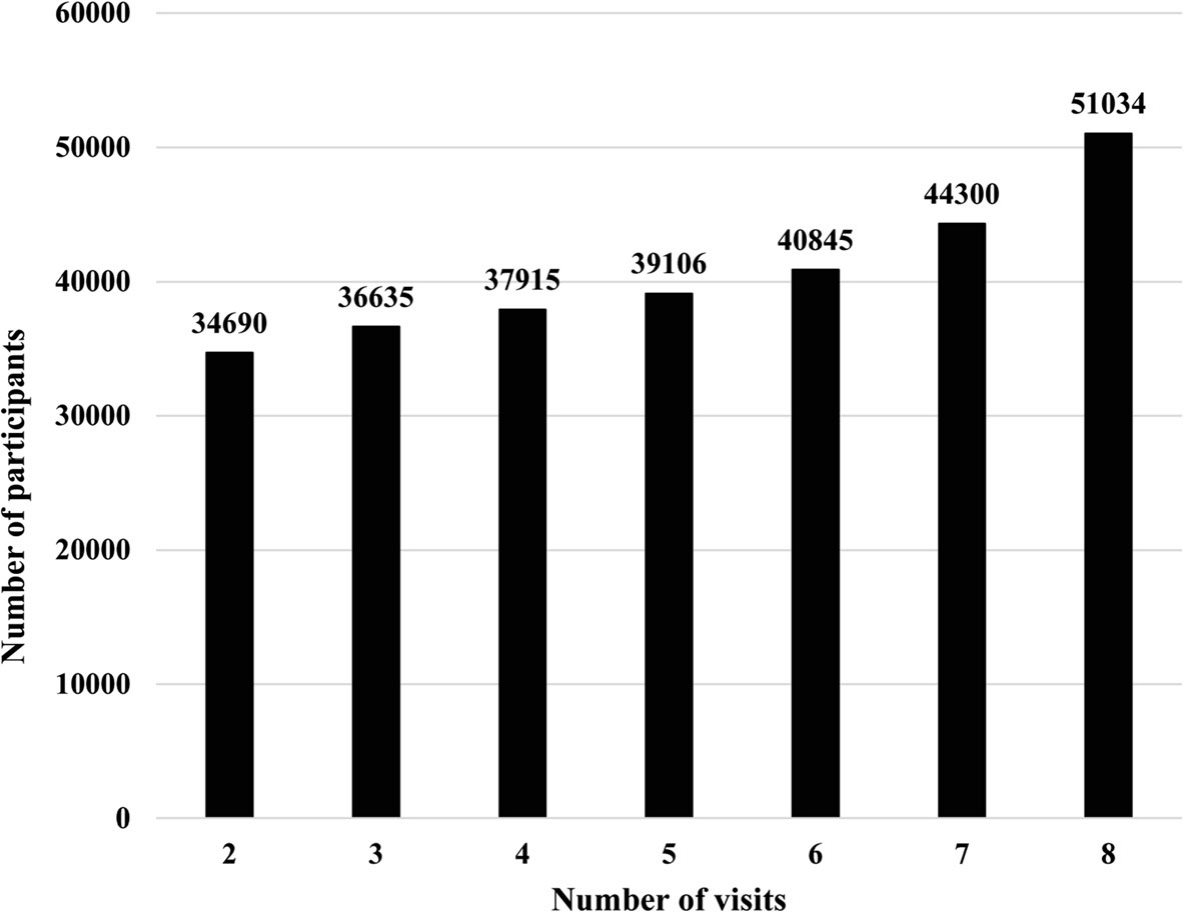
The frequency distribution of number of visits in the Taipei Geriatric Health Examination Database during 2005–2012

A study cohort consisting of participants aged 65 and older was obtained from the Taipei Geriatric Health Examination Database. Our study period covered 2005 to 2012. The Taipei City Government provides free annual health examinations for the elderly population of Taipei City. Each year approximately 40,000 to 46,000 people participate in this program, with approximately a 13 to 16% participation rate [24, 25]. The examination includes a standardized medical examination and questionnaire that addresses a variety of health-related topics. The health examination database we used here is longitudinal and based on voluntary participation by qualified aged citizens in Taipei City. Our study’s purpose is to measure the association between mood disorders and health-related behaviors by repeated measurements, rather than one-shot measurement. Thus, we selected only the participants who came to the health examination program at least two times. Figure 1 shows the overall frequency of the health examination.

### Selection of participants

**Fig. 2:**
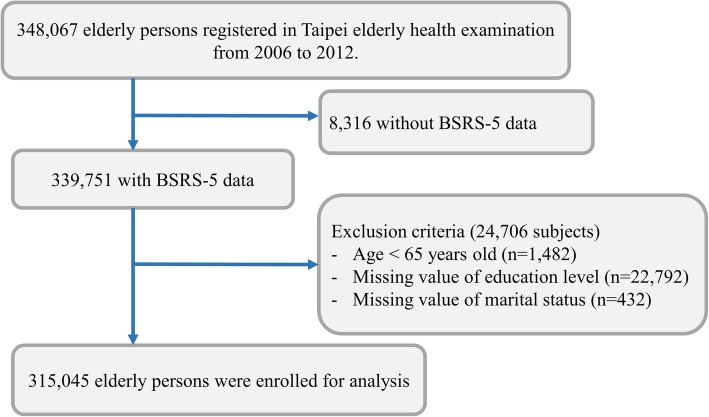
Flow chart of enrollment of study participants

From 348,067 older adults who participated in the elderly health examination in Taipei City, we enrolled 339,751 elderly individuals with mood disorders data. Those with age less than 65 years old or missing data of education level or marital status were excluded. Finally, we enrolled 315,045 study subjects for further analyses (Fig. 2).

### Outcomes

We applied the measurement from BSRS-5 developed by Lung and others [22] as our study outcome. There are five questions in total and each question is from 0 to 4 points. The total score is thus from 0 to 20 points. The questions are as follows: (1) having trouble falling asleep (insomnia); (2) feeling tense or keyed up (anxiety); (3) feeling annoyed easily or irritated (hostility); (4) feeling low mood (depression); and (5) feeling inferior to others (interpersonal hypersensitivity: inferiority). The participants were asked to rate symptoms on a 5-point scale: 0, not at all; 1, a little bit; 2, moderately; 3, quite a bit; and 4, extremely, and a total score was calculated for each participant. The total scores were classified into four levels. Total score > =15 means severe mood disorder, with professional counseling or psychiatric treatment recommended, between 10 and 14 means moderate mood disorder, between 6 and 9 means mild mood disorder, between 0 and 5 means normal group, and these were coded from 4 to 1, respectively.

### Demographic variables, health behaviors and biochemical data

The data for this study include responses to a self-report questionnaire containing demographic variables and health behaviors. One past study using the BSRS-5 questionnaire to screen for psychologic disorders in Taiwan found that it had good reliability and validity [16]. Demographic information, such as age (in five-year increments starting from 65 years old), gender (male and female), BMI, educational level (illiterate, self-taught, elementary school, junior high and senior high school, vocational school, university, and master’s and above), and marital status (married, living together or not; unmarried, widowed, divorced or cohabitating) was collected. For health behaviors, firstly, nutrient data (at least three servings of vegetables and two servings of fruits; and milk drinking), and substance usage (alcohol drinking frequency -- occasionally, daily, or none; and smoking frequency -- after meals, daily or none) were also defined. Secondly, frequency of physical activity (occasional, three to five times per week or even never) was classified. Individual health information was also measured and collected, for example, taking long-term medication or not, number of chronic diseases, and physical and biochemical values (height (cm), weight (kg), systolic and diastolic pressure (mmHg), pulse rate, glucose level (mg/dL), cholesterol level (mg/dL), triglycerides (mg/dL), GOT (U/L), and GPT (U/L)). In this study, we used a two-stage approach to select the potential risk factors including variable selection by logistic regression at the first stage and model fitting by generalized estimating equations (GEE) at the second stage.

### Statistical analyses

Our analysis can be divided into two parts. The first part is to understand the spatio-temporal pattern of mood disorder prevalence in 12 districts of Taipei City. For this, we used the ring map toolbox in ArcGIS 10.2 (Esri Inc., Redlands, CA, USA) [26]. The second part is to identify the risk factors associated with mood disorder. The Chi-square test of independence was used to determine whether there is a significant relationship between mood disorder and personal characteristic variables. The biochemical difference between those with and without mood disorder was statistically analyzed with one-way analysis of variance (ANOVA). We used logistic regression to select the variables with a significance level at *p* < 0.05. To take the within-subject correlation into account, the GEE [27] with a logit link function were conducted for estimating relative risks of the demographic and biochemical factors. All analyses were performed using SPSS version 25.0. Statistical significance was set at *p* < 0.05.

## Results

### Characteristics of the study population

**Fig. 3:**
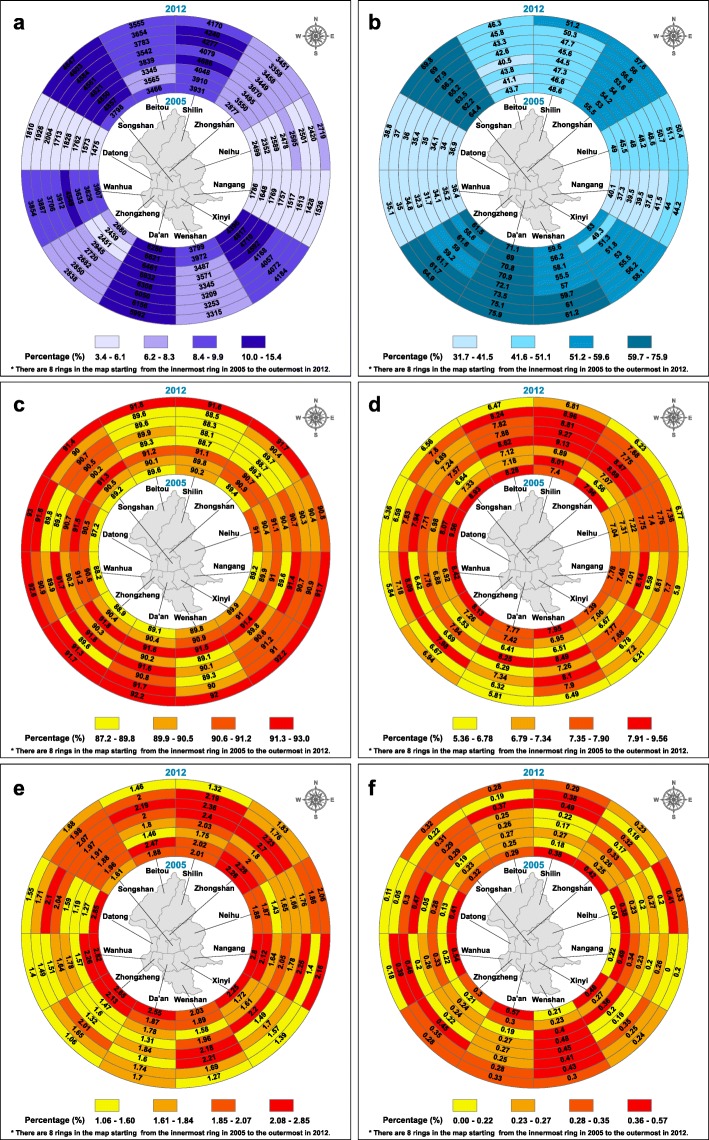
Health examinations, education and mood disorder among the study population in ring map. **a** Frequency of elderly health examination from 2005 to 2012 among Taipei residents. The labels in the rings reprsent the number of participants. **b** Percentage of senior high school and above in Taipei elderly health examination. **c** Percentage of normal mood status from 2005 to 2012 in Taipei elderly health examination. **d** Percentage of mild mood disorders from 2005 to 2012 in Taipei elderly health examination. **e** Percentage of moderate mood disorders from 2005 to 2012 in Taipei elderly health examination. **f** Percentage of severe mood disorders from 2005 to 2012 in Taipei elderly health examination

Data from 2005 to 2012 on a total of 315,045 elderly persons aged over 65 were included in this study. Figure 3a represents the number of elderly persons taking the health examination each year in 12 districts. Higher numbers used the health examination in Da’an, Songshan and Shilin districts. In addition, the percentage of education status higher than senior high school was also high in Da’an and Songshan districts, but low in Shilin (Fig. 3b). The annual percentages of BSRS-5 levels, including normal (Fig. 3c), mild (Fig. 3d), moderate (Fig. 3e), and severe emotional disorders (Fig. 3f) are depicted in four ring maps. Among our study subjects, a high percentage of mild and moderate emotional disorders is found for those living in Shilin (a northern district) (Fig. 3d, e). Notably, there was an increasing trend in moderate emotional disorders from 2005 to 2012 in Nangang District (a southeastern district) (Fig. 3e). Higher percentages (≧0.28%) of elderly persons with serious emotional distress problems were found in Da’an, Wenshan, Shilin, Wanhua and Neihu Districts.

Participants’ characteristics for the groups with or without mood disorder based on BSRS-5 score are shown in Table 1. Most participants were 70–74 years old (25.9%), with elementary school education level (23.6%), female (52.4%), drinking milk (52.0%), eating at least three servings of vegetables and two servings of fruits (77.3%), taking long-term medication (74.3%), non-smoking (94.6%), not drinking alcohol (80.5%), with BMI within 18.5–23.9 (46.5%), self-reporting chronic diseases (43.2%), and not living alone (93.3%). In our results, all variables were significantly different between groups with and without mood disorders except the number of chronic diseases.

**Table 1:** Characteristics of elderly adults with and without mood disorder. Values are counts (percentages) unless stated otherwise

Variable	Total		Mood disorder				χ^2^	*p*-value
			No		Yes^a^			
Age (*n* = 315,045)							51.74	.001
65–69	78,764	(25.0)	71,099	(22.6)	7665	(2.4)		
70–74	81,746	(25.9)	73,729	(23.4)	8017	(2.5)		
75–79	75,425	(23.9)	68,253	(21.7)	7172	(2.3)		
80–84	51,844	(16.5)	47,243	(15)	4601	(1.5)		
> 85	27,266	(8.7)	24,859	(7.9)	2407	(0.8)		
Education (*n* = 306,895)							284.40	.001
Illiterate	17,036	(5.4)	15,116	(4.8)	1920	(0.6)		
Self-taught	6848	(2.2)	6156	(2)	692	(0.2)		
Elementary school	74,255	(23.6)	66,635	(21.2)	7620	(2.4)		
Junior high school	46,211	(14.7)	41,649	(13.2)	4562	(1.4)		
Senior high school	72,394	(23.0)	65,553	(20.8)	6841	(2.2)		
Vocational school	27,442	(8.7)	25,017	(7.9)	2425	(0.8)		
University	62,709	(19.9)	57,517	(18.3)	5192	(1.6)		
Master’s degree and above	8150	(2.6)	7540	(2.4)	610	(0.2)		
Gender (*n* = 315,045)							1285.19	.001
Male	149,920	(47.6)	132,766	(42.1)	17,154	(5.4)		
Female	165,125	(52.4)	152,417	(48.4)	12,708	(4)		
Marital status (*n* = 315,045)							144.45	.001
Unmarried	14,182	(4.5)	12,877	(4.1)	1305	(0.4)		
Cohabitating	1357	(0.4)	1224	(0.4)	133	(0)		
Married–living together	233,800	(74.2)	212,377	(67.4)	21,423	(6.8)		
Married–living separately	6581	(2.1)	5949	(1.9)	632	(0.2)		
Divorced	4059	(1.3)	3634	(1.2)	425	(0.1)		
Widowed	55,066	(17.5)	49,122	(15.6)	5944	(1.9)		
Living alone (*n* = 315,045)							9.40	.002
Yes	20,987	(6.7)	18,872	(6)	2115	(0.7)		
No	294,058	(93.3)	266,311	(84.5)	27,747	(8.8)		
Drinking milk (*n* = 273,621)							146.69	.001
Yes	142,323	(52.0)	129,510	(47.3)	12,813	(4.7)		
No	131,298	(48.0)	117,680	(43.0)	13,618	(5.0)		
Eating at least three servings of vegetables and two of fruits (*n* = 273,343)							1428.72	.001
Yes	211,346	(77.3)	193,371	(70.7)	17,975	(6.6)		
No	61,997	(22.7)	53,562	(19.6)	8435	(3.1)		
Long-term medication (*n* = 315,045)							1140.09	.001
Yes	234,157	(74.3)	209,537	(66.5)	24,620	(7.8)		
No	80,888	(25.7)	75,646	(24.0)	5242	(1.7)		
Smoking status (*n* = 309,030)							9.66	.008
Non-smoking	292,405	(94.6)	264,580	(85.6)	27,825	(9.0)		
Daily smoking	3965	(1.3)	3561	(1.2)	404	(0.1)		
Smoking after a meal	12,660	(4.1)	11,547	(3.7)	1113	(0.4)		
Alcohol frequency (*n* = 314,285)							121.67	.001
Non-drinking	253,083	(80.5)	228,385	(72.7)	24,698	(7.9)		
Daily drinking	6743	(2.2)	6166	(2.0)	577	(0.2)		
Occasional drinking	54,459	(17.3)	49,952	(15.9)	4507	(1.4)		
The frequency of physical activity (*n* = 261,870)							46.38	.001
Never	30,683	(11.8)	27,531	(4.9)	3152	(6.9)		
Three to five times per week	142,049	(54.2)	129,181	(30.7)	12,868	(23.5)		
Occasionally	89,138	(34)	80,703	(16.2)	8435	(17.8)		
Body Mass Index (*n* = 313,262)							33.36	.001
< 18.5	13,006	(4.2)	11,733	(3.7)	1273	(0.4)		
18.5–23.9	145,737	(46.5)	131,764	(42.1)	13,973	(4.5)		
24.0–26.9	98,629	(31.5)	89,651	(28.6)	8978	(2.9)		
27.0–29.9	40,893	(13)	36,990	(11.8)	3903	(1.2)		
30.0–34.9	13,489	(4.3)	12,092	(3.9)	1397	(0.4)		
≥ 35.0	1508	(0.5)	1352	(0.49)	156	(0.01)		
Number of chronic diseases (*n* = 315,018)							15.41	.118
0	93,010	(29.5)	84,423	(26.8)	8587	(2.7)		
1	136,210	(43.2)	123,289	(39.1)	12,921	(4.1)		
2	60,962	(19.4)	55,014	(17.5)	5948	(1.9)		
3	19,629	(6.2)	17,718	(5.6)	1911	(0.6)		
4	4295	(1.4)	3891	(1.2)	404	(0.1)		
≥ 5	912	(0.3)	822	(0.2)	90	(0.1)		

^a^The BSRS-5 scores larger than 5 are defined as participants with mood disorder

Physical and biochemical values of the study population are summarized in Table 2. The results reveal that the averages of height, weight, diastolic blood pressure, total cholesterol, and triglycerides were statistically significant between groups with and without mood disorder.

**Table 2:** Physical and biochemical values of study population

Variable	Reference value	Mean	Mood Disorder		*p*-value
			No	Yes	
Height	-- cm	158.73	158.86	157.48	.001
Weight	-- kg	60.91	61.01	59.93	.001
Systolic	< 120 mmHg	134.61	134.62	134.44	.118
Diastolic	< 80 mmHg	75.16	75.19	74.87	.001
Pulse rate	60–80 /min	73.15	73.15	73.20	.462
Glucose	70–100 mg/dL	104.63	104.64	104.54	.515
Cholesterol	< 200 mg/dL	194.61	194.42	196.43	.001
Triglycerides	< 150 mg/dL	120.41	120.28	121.71	.001
GOT*	8–31 U/L	24.97	24.97	24.99	.795
GPT*	0–41 U/L	22.51	22.52	22.44	.457

*GOT Glutamic Oxaloacetic Transaminase. *GPT* Glutamic Pyruvic Transaminase

People with mood disorders had relatively low height (157.48 vs. 158.86 cm), weight (59.93 vs. 61.01 kg), and diastolic pressure (74.87 vs. 75.19 mmHg), while cholesterol (196.43 vs. 194.42 mg/dL) and triglycerides (121.71 vs.120.28 mg/dL) were relatively high.

The risk factors related to mood disorder are examined with a generalized estimation equation model, and the results are shown in Table 3. People with lower education level, or who were female, divorced, didn’t drink milk, didn’t eat three servings of vegetables and two servings of fruits, or who were taking long-term medication, reported smoking daily, never had physical activity, or had higher cholesterol were all at higher risk of mood disorders than their counterparts. For example, people with a higher educational level revealed a protective effect (both OR and 95% CI of university and master’s degree or above are less than 1). In terms of gender, males had a very low OR (0.572) compared to females. In marital status, the divorced had a significantly higher OR (1.22) compared to married--living together. As for healthy behaviors, not drinking milk (1.117), not eating three servings of vegetables and two servings of fruits (1.782) and daily smoking (1.152) all had significantly high OR compared to their counterparts. However, people not taking long-term medication (0.571), occasionally drinking (0.955), occasionally exercising (0.955), aged between 80 and 85, and those with fewer chronic diseases had significantly lower risk of mood disorder. Regarding biochemical values, people with higher cholesterol had a higher odds ratio related to mood disorder.

**Table 3:** Risk factors of mood disorder

Variable	Beta (95% CI)	*p*-value	OR (95% CI)
Demographic variables			
Education			
Master’s and above	−0.129 (−0.2 to −0.06)	.001	0.879 (0.82 to 0.95)
University	−0.077 (− 0.11 to − 0.04)	.001	0.926 (0.89 to 0.96)
Vocational school	− 0.044 (− 0.09 to 0)	.051	0.957 (0.92 to 1)
Junior high school	−0.025 (− 0.06 to 0.01)	.147	0.976 (0.94 to 1.01)
Senior high school	−0.019 (− 0.06 to 0.02)	.324	0.981 (0.95 to 1.02)
Self-taught	− 0.039 (− 0.12 to 0.04)	.341	0.962 (0.89 to 1.04)
Illiterate	0.025 (−0.03 to 0.08)	.363	1.025 (0.97 to 1.08)
Elementary school			
Gender			
Male	−0.559 (−0.59 to − 0.53)	.001	0.572 (0.56 to 0.59)
Female			
Marital status			
Widowed	0.026 (−0.01 to 0.06)	.125	1.027 (0.99 to 1.06)
Divorced	0.199 (0.09 to 0.31)	.001	1.22 (1.09 to 1.36)
Married--living separately	0.004 (−0.08 to 0.08)	.931	1.004 (0.93 to 1.09)
Cohabitating	−0.107 (− 0.28 to 0.07)	.222	0.899 (0.76 to 1.07)
Unmarried	−0.027 (− 0.08 to 0.03)	.328	0.974 (0.92 to 1.03)
Married--living together			
Drinking milk			
No	0.111 (0.09 to 0.13)	.001	1.117 (1.09 to 1.14)
Yes			
Eating at least three servings of vegetables and two of fruits			
No	0.578 (0.55 to 0.61)	.001	1.782 (1.73 to 1.83)
Yes			
Long-term medication			
No	−0.561 (−0.58 to −0.54)	.001	0.571 (0.56 to 0.58)
Yes			
Smoking frequency			
Smoking after meals	−0.034 (−0.09 to 0.02)	.193	0.966 (0.92 to 1.02)
Daily smoking	0.142 (0.01 to 0.28)	.041	1.152 (1.01 to 1.32)
Non-smoking			
Alcohol frequency			
Occasionally drinking	−0.046 (−0.08 to − 0.02)	.002	0.955 (0.93 to 0.98)
Daily drinking	−0.067 (− 0.15 to 0.01)	.098	0.935 (0.86 to 1.01)
Non-drinking			
The frequency of physical activity			
Occasionally	−0.046 (−0.09 to − 0.01)	.024	0.955 (0.92 to 0.99)
Three to five times per week	−0.059 (− 0.1 to − 0.02)	.002	0.942 (0.91 to 0.98)
Never			
Age			
> 85	−0.032 (− 0.08 to 0.01)	.160	0.968 (0.93 to 1.01)
80–84	−0.042 (− 0.08 to − 0.01)	.022	0.959 (0.93 to 0.99)
75–79	− 0.008 (− 0.04 to 0.03)	.650	0.993 (0.96 to 1.03)
70–74	0.001 (− 0.03 to 0.03)	.940	1.001 (0.97 to 1.03)
65–69			
Number of chronic diseases	0.021 (0.01 to 0.03)	.002	1.02 (1.01 to 1.03)
Biochemical value			
Cholesterol	0.001 (0.001–0.001)	.014	1.00 (1.00–1.00)

Furthermore, we tried to examine the risk differences between genders. There appeared no risk differences between genders in educational level, milk drinking, vegetables and fruits intake, long-term medication, and cholesterol level, while marital status, smoking and alcohol frequency, exercise frequency, age, and the number of chronic diseases presented mild variations between males and females (Table 4).

**Table 4:** Risk factors of mood disorder of gender

Variables	OR (95% CI)	
	Male	Female
Demographic variables		
Education		
Master’s and above	0.893 (0.82 to 0.97)*	0.868 (0.71 to 1.06)
University	0.938 (0.9 to 0.98)*	0.91 (0.84 to 0.98)*
Vocational school	0.958 (0.91 to 1.02)	0.962 (0.88 to 1.05)
Junior high school	0.983 (0.94 to 1.03)	0.966 (0.9 to 1.03)
Senior high school	1.009 (0.95 to 1.07)	0.958 (0.9 to 1.03)
Self-taught	0.926 (0.82 to 1.04)	0.992 (0.93 to 1.06)
Illiterate	1.071 (0.97 to 1.19)	0.979 (0.87 to 1.1)
Elementary school		
Marital status		
Widowed	1.069 (1.01 to 1.13)*	1.006 (0.97 to 1.05)
Divorced	1.386 (1.19 to 1.61)*	1.063 (0.91 to 1.24)
Married--living separately	1.026 (0.93 to 1.13)	0.982 (0.86 to 1.12)
Cohabitating	0.997 (0.77 to 1.3)	0.852 (0.68 to 1.07)
Unmarried	1.026 (0.96 to 1.1)	0.91 (0.84 to 0.99)*
Married--living together		
Drinking milk		
No	1.111 (1.08 to 1.14)*	1.125 (1.09 to 1.16)*
Yes		
Eating at least three servings of vegetables and two of fruits		
No	1.69 (1.63 to 1.76)*	1.885 (1.8 to 1.97)*
Yes		
Long-term medication		
No	0.606 (0.59 to 0.63)*	0.538 (0.52 to 0.56)*
Yes		
Smoking frequency		
Smoking after meals	0.981 (0.93 to 1.04)	0.902 (0.79 to 1.03)
Daily smoking	1.179 (1.02 to 1.37)*	1.026 (0.71 to 1.48)
Non-smoking		
Alcohol frequency		
Occasionally drinking	0.956 (0.93 to 0.99)*	0.956 (0.9 to 1.01)
Daily drinking	0.981 (0.9 to 1.07)	0.719 (0.57 to 0.91)*
Non-drinking		
Frequency of physical activity		
Occasional	0.978 (0.92 to 1.04)	0.942 (0.89 to 1)
Three to five times per week	0.966 (0.92 to 1.02)	0.928 (0.88 to 0.98)*
Never		
Age		
> 85	0.964 (0.91 to 1.02)	0.98 (0.91 to 1.06)
80–84	0.949 (0.91 to 0.99)*	0.985 (0.93 to 1.05)
75–79	0.988 (0.95 to 1.03)	0.999 (0.95 to 1.05)
70–74	1.012 (0.97 to 1.06)	0.993 (0.95 to 1.04)
65–69		
Number of chronic diseases	1.008 (0.99 to 1.02)	1.029 (1.01 to 1.05)*
Biochemical value		
Cholesterol	1 (1 to 1)	1 (1 to 1)

* *p*-value <0.05

## Discussion

This study applied long-term follow-up data on the elderly participating in the Taipei health examination at least twice to analyze the possible risk factors for mood disorders in the elderly. In our study, we collected all potential risk factors from the health examination. In addition to finding out the risk factors from the existing literature, we also tried to examine some new factors from our unique database. Although elderly mental health is an important issue in past studies, this study demonstrated different kinds of potential risk factors together and had multiple repeated measurements to validate the results. We found not only risk factors, but also protective factors for mood disorders in the elderly simultaneously in one study. The results suggest that health policy makers implement behavior intervention to reduce mood disorders with health education. We found that the residents living in districts with a higher average educational level were more willing to participate in the health examination. In addition, we also identified the personal demographic factors, health behaviors and self-report of medication factors associated with mood disorders.

Our results reveal that the demographic variables of female, older age, lower education level, and divorced status are associated with mood disorders. Specifically, previous studies have shown that gender [28, 29] and age [30] are associated with depression. The results in the present study are consistent with domestic and international research, which indicated that gender difference also plays a role in mental health [31, 32]. For the elderly, our study illustrates that marital status (particularly, divorce) is a key factor associated with mood disorders. One study in Singapore found that females have higher depressive symptom scores than males. However, living alone and weak social networks outside the household were associated with higher depressive symptoms in both males and females [29]. Hence, for the elderly, living alone, not being married [18], and weak social networks may all increase their emotional isolation and social disengagement. They are consequently more likely to have mood disorders. Higher education seems to be a protective factor in the elderly [33]. A previous study showed that higher social activity was associated with a lower risk of late-life depressive symptoms at a baseline in a large community sample with 2 years of follow-up [34]. Another study, in Korea, founded that those who participated in frequent physical, social, and some religious activity had a low odds ratio for depression compared with their counterparts [35]. In summary, these elderly with few social network links and/or who are divorced may have high risk of mood disorders because they are lonely and seldom interact with others.

The percentage of elderly people who participated in the health examination in this study who were living in Datong, Nangang and Neihu districts is low. The proportion with a high school education and above in these areas is low as well (less than 50%). The above results lead us to speculate that education level may affect health risk awareness. People with higher education level are more concerned about their health and willing to conduct regular health examinations [36–38]**.** In addition, we found that those with lower education levels were more likely to suffer from mood disorders, which was consistent with results of previous studies [30, 39, 40]. Low economic status may be associated with educational opportunity and less chance to access medical service [41]. The worst geographical areas in terms of mood disorders are Wenshan, Shilin and Zhongshan districts. In contrast, the areas with the highest proportion of normal mood status are Nangang, Xinyi, Da’an and Zhongzheng districts. Among them, people in Xinyi and Da’an districts had senior high school education level in a higher proportion than other districts. These areas are important business and economic areas. Therefore, the better mood may be owing to having a better material life for the elderly [42, 43]. In contrast, low education level and economic status may elevate life pressure, which may directly or indirectly worsen mood status.

Additionally, lifestyle is the most important factor affecting health [44]. Healthy behaviors, for example, food and alcohol intake, smoking and frequency of physical activity, are included. Our study also revealed the lifestyle characteristics of the elderly that may be associated with mood disorders. We found that drinking milk, as well as eating at least three servings of vegetables and two servings of fruits, is associated with a lower chance of developing mood disorders in the elderly. The present study found that elderly people who had no depressive symptoms were more likely to consume well-balanced meals and milk products on a daily basis [45]. In addition, one study illustrated that adherence to a Mediterranean dietary pattern ensures an adequate intake of fruits, nuts, vegetables, cereals, legumes and fish, all of which are important sources of nutrients linked to lower risk of depression [46]. Another study indicated that intake of milk products is also thought to be related to reduced depressive symptoms among elderly people [47]. Calcium and milk intake is indirectly related to depressive symptoms because it prevents central adiposity, which has been associated with depression [48]. This finding supports current recommendations for increasing milk, fruit and vegetable intake to improve mental health. Moreover, Nakao and Yano (2004) determined that depression is associated with high serum cholesterol levels among middle-aged Japanese men [19]. This is similar to our finding that higher cholesterol levels were associated with mood disorders in the elderly.

On the other hand, unhealthy behaviors, for example, daily smoking, and never exercising, were risk factors of mood disorders. Two previous studies have illustrated that people who smoke or have smoked are more depressed than those who have not done so before, but these findings were significant in only one of the two studies [18, 19]. However, occasionally drinking alcohol has a mild protective effect against mood disorders. This result is also similar to a previous study, which indicated that moderate alcohol consumption in women was related to fewer depressive symptoms in later life [48]. Additionally, previous studies have revealed that leisure activity participation can improve psychological well-being in older adults [49]. Similar to our findings, those suggest that participation in recreational physical activity is associated with a lower chance of developing mood disorders among the elderly. The mental happiness achieved through physical activity enables the elderly to reduce their stress.

Furthermore, poor health status has been demonstrated to be a risk factor for depression in the elderly, such as having a history of stroke, cancer or Parkinson’s disease [50, 51]. Patients may feel frustrated because they lose their self-care ability and feel they are troublesome to their families. Among our participants, their self-reported history of medication and chronic disease showed that having more chronic diseases is associated with mood disorders among the elderly.

Previous studies have indicated that there are many risk factors related to depression. From the perspective of preventive medicine, mental health of the elderly in the community is very important. Through community monitoring, risk factors affecting the mood of the elderly can be found, and this will help with follow-up prevention work. Our study has shown that age, education, whether one lives alone, smoking, drinking, vegetable intake, long-term medication and BMI are significantly different in the elderly who have or do not have mood disorders. The results can provide us a micro-level understanding of the differences in mood disorders vis-à-vis the characteristics of elderly people in Taipei.

However, there are some limitations in this study. A major one is that the sample consisted of elderly participants who were willing to take part in the health examination; thus, the sampling was not randomized. The voluntary participants of this study may not be representative of the general population. Our cohort most likely represented a younger and more highly educated population, with more people having intact cognitive abilities. We might underestimate the risk from self-selection bias of healthier participants. Second, it was a second-hand dataset containing self-reported information, which may carry some bias, such as recall bias. Another limitation is that we only use BSRS-5 to estimate the outcome. Other measurements or clinical diagnosis could be added for more precise estimation in the future.

### Policy indication

First, the questionnaire of BSRS-5 is an easy and fast tool to evaluate mental disorders. It is easily deployed in community health examination. Our results indicated that social support, for example, marital status, is a significant factor associated with mental disorders. Policy makers should encourage the elderly to participate in social activities and to reduce loneliness and the risk of mental disorders. Third, this study also found that health behaviors including physical activity, drinking milk, eating more vegetables, and quitting smoking are linked to better mental health. Many of these factors can potentially be improved through health education to reduce the risk of mental disorders. Lastly, the more chronic diseases one suffers from, the higher the risk of mental disorders in the elderly, suggesting that the health system needs to control and reduce chronic diseases in the elderly.

## Conclusion

Our study provides a new approach for better understanding of mood disorders among the elderly with data from a long-term follow-up community health examination. Certain values of demographic variables, such as female gender, low education level and divorced; health behaviors of non-intake of milk, non-intake of fruit and vegetables, smoking and never exercising; and individual variables of long-term medication and a greater number of diseases were all associated with mood disorders. The findings of this research provide a macro view for understanding mood disorders as well as a micro view for identifying risk factors of mood disorders in the elderly through health examination. Our study suggests that health policy makers should focus on reducing those risk factors for developing mood disorders among the elderly.

## Abbreviations

95% CI: 95% confidence interval
ANOVA: Analysis of variance
BMI: Body mass index
BSRS-5: 5-item Brief Symptom Rating Scale
GEE: Generalized estimating equations
NHS: National Health Service
OR: Odds ratio
WHO: World Health Organization
